# Novel variants in *TUBB8* gene cause multiple phenotypic abnormalities in human oocytes and early embryos

**DOI:** 10.1186/s13048-023-01274-3

**Published:** 2023-11-25

**Authors:** Tingwenyi Hu, Chong Li, Sen Qiao, Weiwei Liu, Wei Han, Wei Li, Rong Shi, Xia Xue, Juanzi Shi, Guoning Huang, Tingting Lin

**Affiliations:** 1 Chongqing Key Laboratory of Human Embryo Engineering, Center for Reproductive Medicine, Women and Children’s Hospital of Chongqing Medical University, Chongqing, 400010 China; 2 Chongqing Clinical Research Center for Reproductive Medicine, Chongqing Health Center for Women and Children, Chongqing, 400010 China; 3 Reproductive Center, Northwest Women’s and Children’s Hospital, Xi’an, 710003 Shaanxi China

**Keywords:** Primary female infertility, Oocyte maturation arrest, *TUBB8* gene, Genetic counselling

## Abstract

**Background:**

The genotype-phenotype relationships between *TUBB8* variants and female infertility are difficult to clearly define due to the complex inheritance patterns and the highly heterogeneous phenotypes. This study aims to identify novel *TUBB8* variants and relevant phenotypes in more infertile females.

**Methods:**

A total of 35 females with primary infertility were recruited from two reproductive centers and investigated for identifying variants in *TUBB8*. Pedigree analysis, *in-silico* analysis and molecular remodeling were performed to assess their clinical significance. The effects of the variants on human oocytes and embryos as well as HeLa cells were analyzed by morphological observations, immunostaining and Western blot.

**Results:**

We totally identified five novel variants (p.G13R, p.Y50C, p.T136I, p.F265V and p.T366A) and five previously reported variants (p.I4L, p.L42V, p.Q134*, p.V255M and p.V349I) in *TUBB8* from 9 unrelated females with primary infertility. These variants were rare and highly conserved among different species, and were inherited in autosomal dominant/recessive patterns, or occurred *de novo*. In vitro functional assays in HeLa cells revealed that exogenous expression of mutant TUBB8 proteins caused different degrees of microtubule structural disruption. The existence of these pathogenic *TUBB8* variants finally induced oocyte maturation arrest or morphological abnormalities, fertilization failure, cleavage failure, embryonic development defects and implantation failure in the affected females.

**Conclusion:**

These findings enriched the variant spectrum of *TUBB8* gene and could contribute to optimize genetic counselling and clinical management of females with primary infertility.

**Supplementary Information:**

The online version contains supplementary material available at 10.1186/s13048-023-01274-3.

## Background

Infertility is a disease of reproductive system defined by the failure to achieve a pregnancy after 12 months or more of regular unprotected sexual intercourse [1]. According to the World Health Organization, around 17.5% of the adult population, roughly 1 in 6 worldwide, experience infertility [2]. Assisted reproductive technology (ART) has been widely applied for helping infertile couples to get progenies through in vitro fertilization (IVF) or intracytoplasmic sperm injection (ICSI). However, some couples suffered from repeated IVF and/or ICSI failures due to, for instance, oocyte maturation arrest and early embryonic development defects, and the strategies for improving reproductive outcomes are limited [3, 4].

From oocyte maturation to early embryo formation is a prolonged and highly organized process consisting of germinal vesicle (GV) breakdown, meiosis I, meiosis II, fertilization, cleavage, and early embryonic development, and its developmental block occurs at any stage, such as GV, metaphase I (MI), metaphase II (MII), fertilization, cleavage, and early embryonic development, termed as oocyte/zygote/embryo maturation arrest (OZEMA) [5, 6]. Intriguingly, increasing number of studies have demonstrated the important role of genetic factors in OZEMA, such as *TUBB8*, *PATL2*, *ZP1*, *ZP2*, *ZP3*, *CDC20*, *TRIP13* and *BTG4* [7]. The *TUBB8* gene (MIM: 616,768), which is located at chromosome 10p15.3 and encodes a predicted 444-amino acid protein, had been firstly described of genotype-phenotype relationship with OZEMA (OZEMA2, MIM: 616,780) in 2016 [8]. TUBB8 protein is a primate-specific isotype of β-tubulin, forming a heterodimer with α-tubulin to constitute the error-prone spindle of oocyte and early embryo [9, 10]. To date, more than 100 variants in *TUBB8* gene have been identified and related to approximately 30% of infertile females with OZEMA in complex inheritance patterns, such as autosomal dominantly inheriting from father or arising *de novo*, or autosomal recessively inheriting from father and mother, or inheriting from mother with incomplete penetrance [11–13]. Subsequently, the disruption of microtubule formation and spindle assembly in oocyte and early embryo induced repeated ART failures with highly heterogeneous phenotypes, including GV arrest, MI arrest, oocytes with abnormal morphology, fertilization failure, cleavage failure, early embryonic development arrest and implantation failure [12–18]. These studies further highlight the essential role of TUBB8 in oocyte maturation as well as early embryonic development and demonstrated the necessity of genetic counseling for high genetic and phenotypic heterogeneity of *TUBB8* variants and disease phenotypes. Consequently, further verification of phenotypes exhibited by the reported *TUBB8* variants and identification of novel *TUBB8* variants remain necessary.

In our previous study, we had identified 11 *TUBB8* variants from 15 unrelated families with complicated inheritance patterns and variable phenotypes [13]. In this study, we focused on continuously recruiting females with primary infertility from two reproductive centers to detect *TUBB8* variants and phenotypes. Through Sanger sequencing, nine missense variants and one loss-of-function variant, including five novel variants and five previously reported variants, were identified in nine unrelated families. In vitro functional assays with HeLa cells expressing wild-type or mutant TUBB8 proteins were performed to assess their effect on protein stability and microtubule structure. The phenotypes caused by these *TUBB8* variants and the outcomes of IVF or ICSI were investigated. These findings enriched the variant spectrum of *TUBB8* gene and could contribute to optimize the genetic counselling and clinical management of infertile females.

## Results

### **Identification of *TUBB8 *variants from infertile females with OZEMA**

**Fig. 1:**
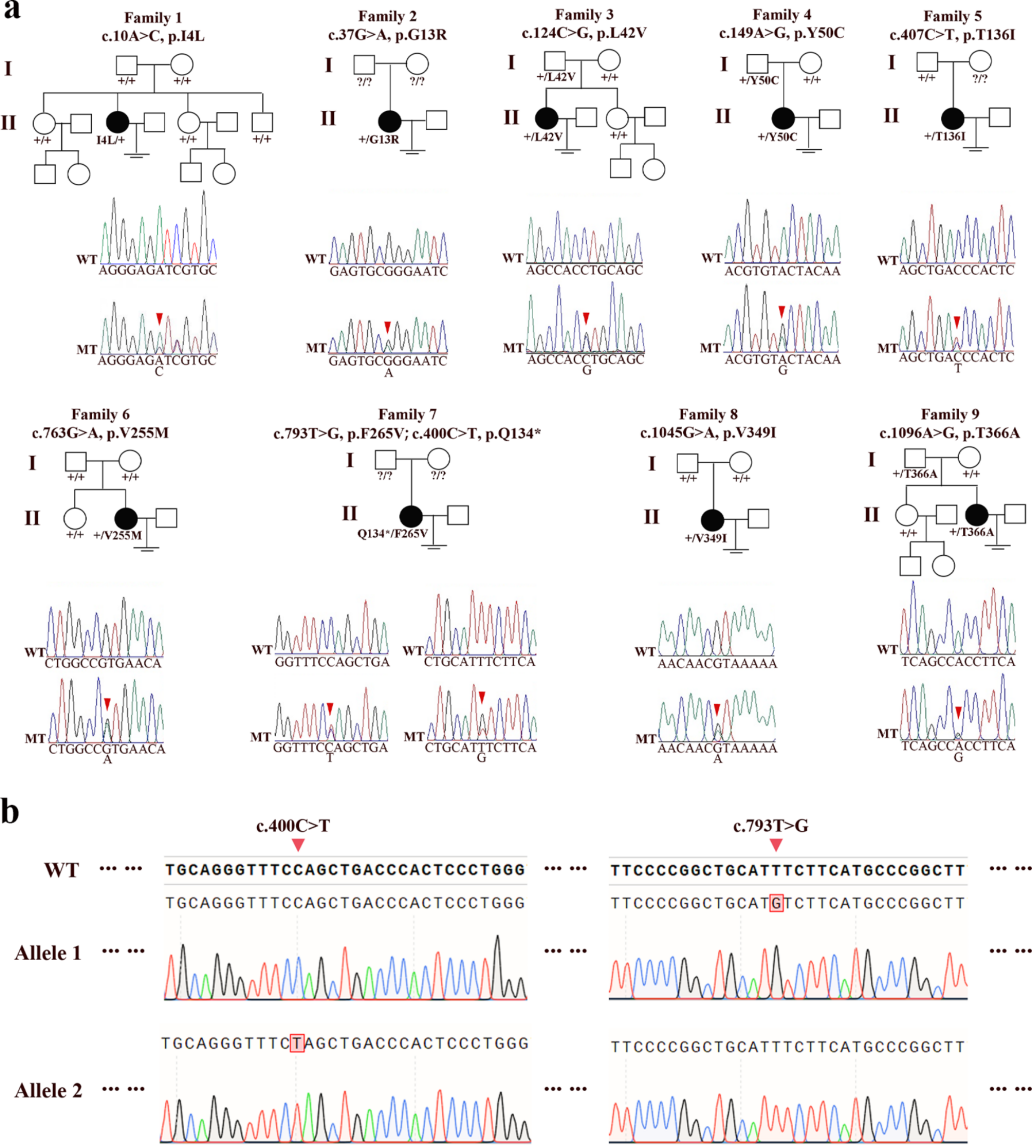
**Pedigrees and*TUBB8*variants of 9 families. a** Pedigrees and Sanger sequencing chromatograms of 9 families. The “〧” sign indicates infertility, the black circles represent affected individuals, the question marks indicate absence of DNA samples, ‘+’ means wild-type, and the red arrows indicate mutation loci. MT, mutant allele; WT, wild-type allele. **b** TA cloning of Family 7, the red arrows indicate mutation loci; WT, wild-type allele

We identified nine missense and one loss-of-function variants of *TUBB8* from nine unrelated females, including five novel variants (c.37G > A, p.G13R; c.149 A > G, p.Y50C; c.407 C > T, p.T136I; c.793T > G, p.F265V; c.1096 A > G, p.T366A) and five previously reported variants (c.10 A > C, p.I4L; c.124 C > G, p.L42V; c.400 C > T, p.Q134*; c.763G > A, p.V255M; c.1045G > A, p.V349I) [11, 19–22] (Fig. 1a). These variants presented as heterozygous state in all families, while two of them (c.400 C > T, p.Q134*; c.793T > G, p.F265V) coexisted in the proband of Family 7 (Table 1). Variants in Family 1, 6 and 8 occurred *de novo*, while variants in Family 3, 4 and 9 were inherited from the fathers. The variants’ inheritance pattern in Family 2, 5 and 7 were unknown due to the unavailability of DNA samples from their parents. TA cloning revealed that the two heterozygous *TUBB8* variants of Family 7 were *in trans*, indicating that it was inherited probably in an autosomal recessive pattern (Fig. 1b). *In silico* analysis indicated that almost all the variants disrupted the function of TUBB8, as predicted by PolyPhen-2, SIFT, MutationTaster, and CADD, and they all have rare frequencies or were absent in the gnomAD database (Table 1). Thus, we speculated that these likely pathogenic variants of *TUBB8* were the primary cause of OZEMA.

**Table 1:** Details of *TUBB8* variants identified in this study

Family no.	Age	Chromosome localization	Exon/ Intron	cDNA change	Protein change	Mutation zygosity	Inheritance	Known/ novel	Allele frequency		In silico bioinformatics prediction			
									gnomAD	gnomAD-EAS	PolyPhon-2	MutationTaster	SIFT	CADD
Family 1	29	Chr10:95169	Exon 1	c.10 A > C	p.I4L	Het	de novo/AD	Known	0.00000419	0	Benign	Disease-causing	Damaging	Tolerable
Family 2	34	chr10:95142	Exon 1	c.37G > A	p.G13R	Het	NA/AD	Novel	0.00001549	0	Probably-damaging	Disease-causing	Damaging	Damaging
Family 3	30	chr10:94786	Exon 2	c.124 C > G	p.L42V	Het	Father/AD	Known	0.0001	0.0012	Probably-damaging	Disease-causing	Damaging	Tolerable
Family 4	35	chr10:94761	Exon 2	c.149 A > G	p.Y50C	Het	Father/AD	Novel	ND	ND	Probably-damaging	Disease-causing	Damaging	Damaging
Family 5	26	chr10:93925	Exon 4	c.407 C > T	p.T136I	Het	NA/AD	Novel	ND	ND	Probably-damaging	Disease-causing	Damaging	Damaging
Family 6	31	chr10:93569	Exon 4	c.763G > A	p.V255M	Het	de novo/AD	Known	0.00002163	0	Probably-damaging	Disease-causing	Damaging	Damaging
Family 7	30	chr10:93539	Exon 4	c.793T > G	p.F265V	Het	NA/AR	Novel	ND	ND	Probably-damaging	Disease-causing	Damaging	Damaging
		chr10:93932	Exon 4	c.400 C > T	p.Q134*	Het	NA/AR	Known	0	0	NA	Disease-causing	NA	Damaging
Family 8	35	chr10:93287	Exon 4	c.1045G > A	p.V349I	Het	de novo/AD	Known	ND	ND	Probably-damaging	Disease-causing	Damaging	Tolerable
Family 9	29	chr10:93236	Exon 4	c.1096 A > G	p.T366A	Het	Father/AD	Novel	ND	ND	Probably-damaging	Disease-causing	Damaging	Tolerable

Het, heterozygous; AD, autosomal dominant; NA, not available; ND, not detected; AR, autosomal recessive

### **Bioinformatic analysis of *TUBB8 *variants**

**Fig. 2:**
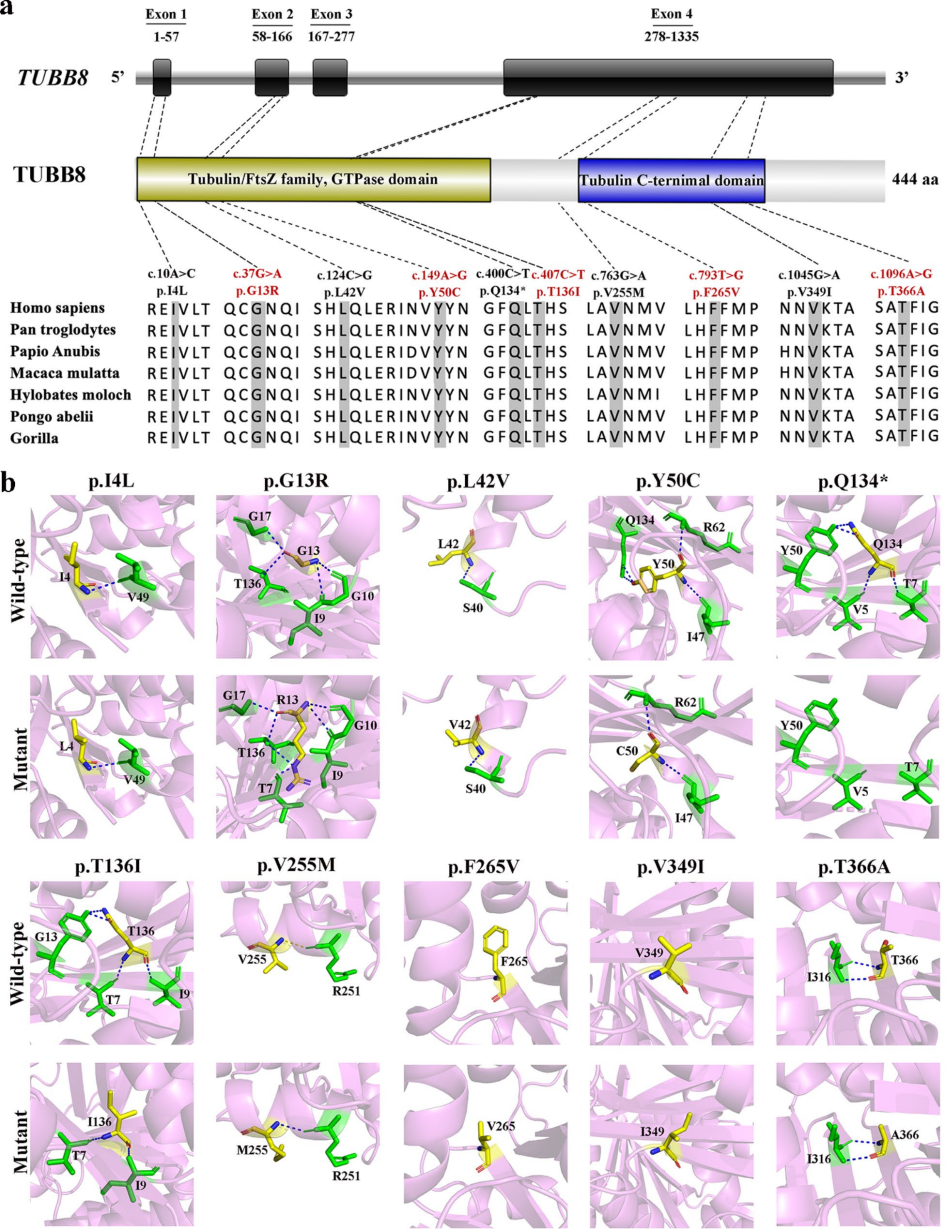
**Bioinformatic analysis of*TUBB8*variants. a** Locations and conservation analysis of the identified variants in TUBB8 protein. The red font indicates novel variants and the black font indicates previously reported variants. **b** Protein conformation predictions of *TUBB8* variants. Blue dashed lines represent hydrogen bonds

*TUBB8* gene (NM_177987.2) is mapped to chromosome 10p15.3 and consists of 4 coding exons. As shown in Fig. 2a, amid the ten variants we identified, two were located in exon 1, two in exon 2, and six in exon 4. The amino acid changes caused by these variants were distributed over the full length of TUBB8 protein. All the ten altered amino acids at variant positions were highly conserved among different species (Fig. 2a). Additionally, we predicted the effect of these variants on the structure of TUBB8 protein using three-dimensional protein structure based on PyMol software (Fig. 2b). The variants p.G13R, p.L42V and p.V255M occurred in α-helix region, while the variants p.I4L, p.Y50C, p.Q134*, p.T136I, p.F265V, p.V349I and p.T366A occurred in β-folding region. The p.Q134* variant caused premature termination of protein translation and suggested its strong deleterious effect. The p.G13R variant added the hydrogen bonds interacting with T136 and T7, which may alter the folding and thus affect microtubule assembly. The variants of p.Y50C and p.T136I resulted in the loss of hydrogen bonds interacting with Q134 and G13, respectively, which could destabilize its folding and thereby affect microtubule stability. Herein, these variants probably disrupted the normal function of TUBB8 protein in microtubules.

### Effect of wild-type and mutant TUBB8 proteins on microtubule network in HeLa cells

**Fig. 3:**
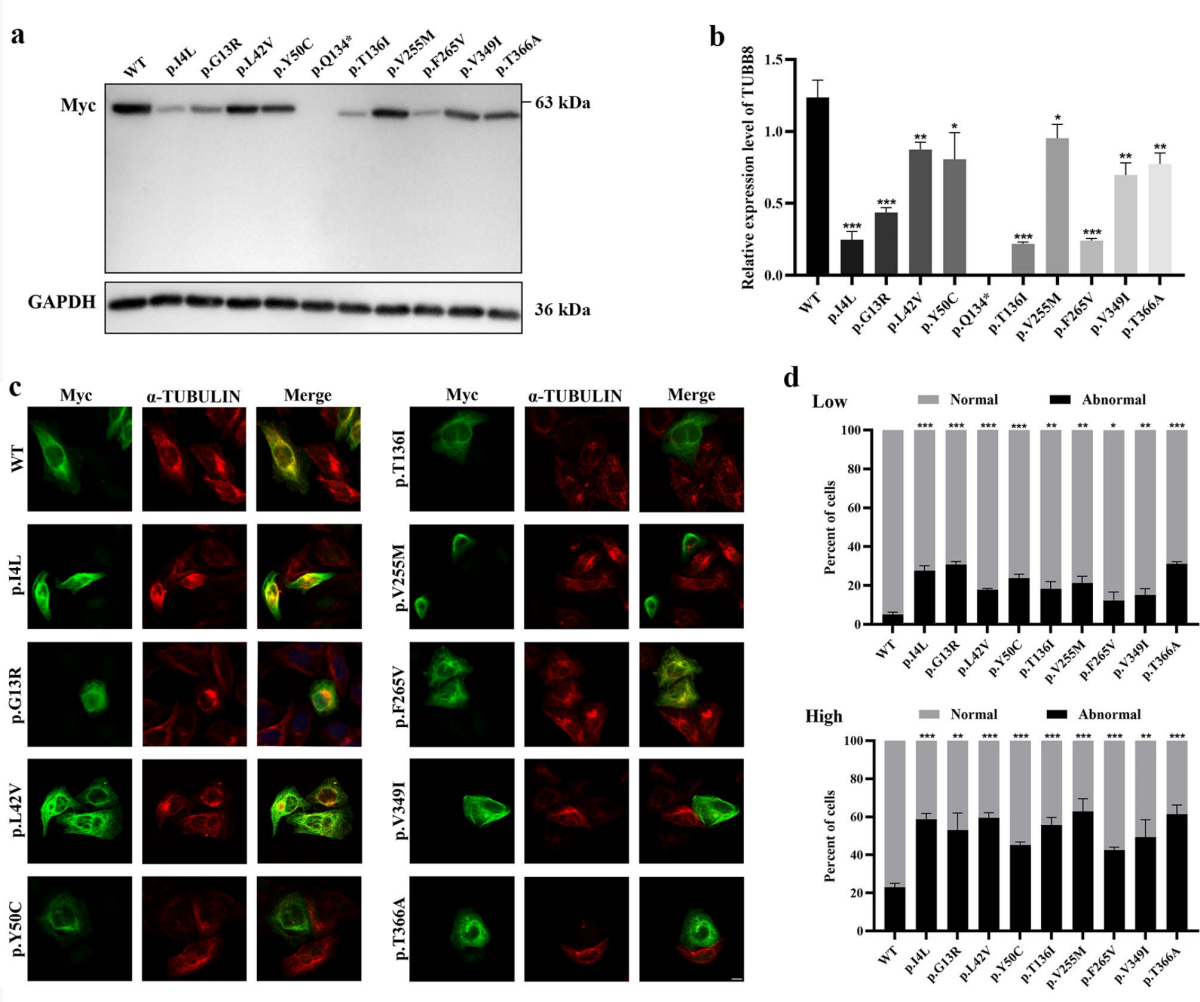
**Expression of wild-type and mutant TUBB8 proteins in HeLa cells. a,b** The protein expression level of TUBB8 in HeLa cells transfected with plasmids encoding Myc-tagged wild-type and mutant TUBB8 proteins. WT, wild-type. * *P* < 0.05, ** *P* < 0.01, *** *P* < 0.001. **c,d** Microtubule phenotypes induced by the expression of wild-type and mutant TUBB8 proteins in HeLa cells. Cells were immunoassayed with an antibody against Myc epitope (green) to visualize transgene and counterstained with an antibody against α-TUBULIN (red) to visualize endogenous microtubule network. Scale bar, 10 μm. WT, wild-type. * *P* < 0.05, ** *P* < 0.01, *** *P* < 0.001

To further investigate the pathogenic potential of the identified *TUBB8* variants, we transfected HeLa cells with plasmids expressing wild-type or mutant TUBB8 proteins and conducted functional assays. Western blot analysis revealed that the expression level of mutant TUBB8 proteins were significantly decreased than that of wild-type TUBB8 (Fig. 3a and b), indicating that these variants induced different degrees of instability of TUBB8 proteins. The variant of p.Q134* resulted in the complete degradation of TUBB8 protein without any truncated protein detected (Fig. 3a). Immunofluorescence assay revealed that when exogenous TUBB8 protein was expressed at a relatively low level, the wild-type and mutant proteins exerted little effects on the microtubule network (Fig. 3c and d). However, when expression at a relatively high level, both wild-type and mutant proteins exerted obvious effects on the microtubule network triggering severe structural damage (Fig. 3c and d). Meanwhile, all the mutant proteins had a significantly greater propensity than the wild-type protein to cause microtubule abnormalities in both low and high expression cases (Fig. 3d). These results suggested that the identified *TUBB8* variants could affect the stability of TUBB8 protein as well as the endogenous microtubule structure.

### **Oocyte and embryo phenotypes of probands harboring *TUBB8 *variants**

**Fig. 4:**
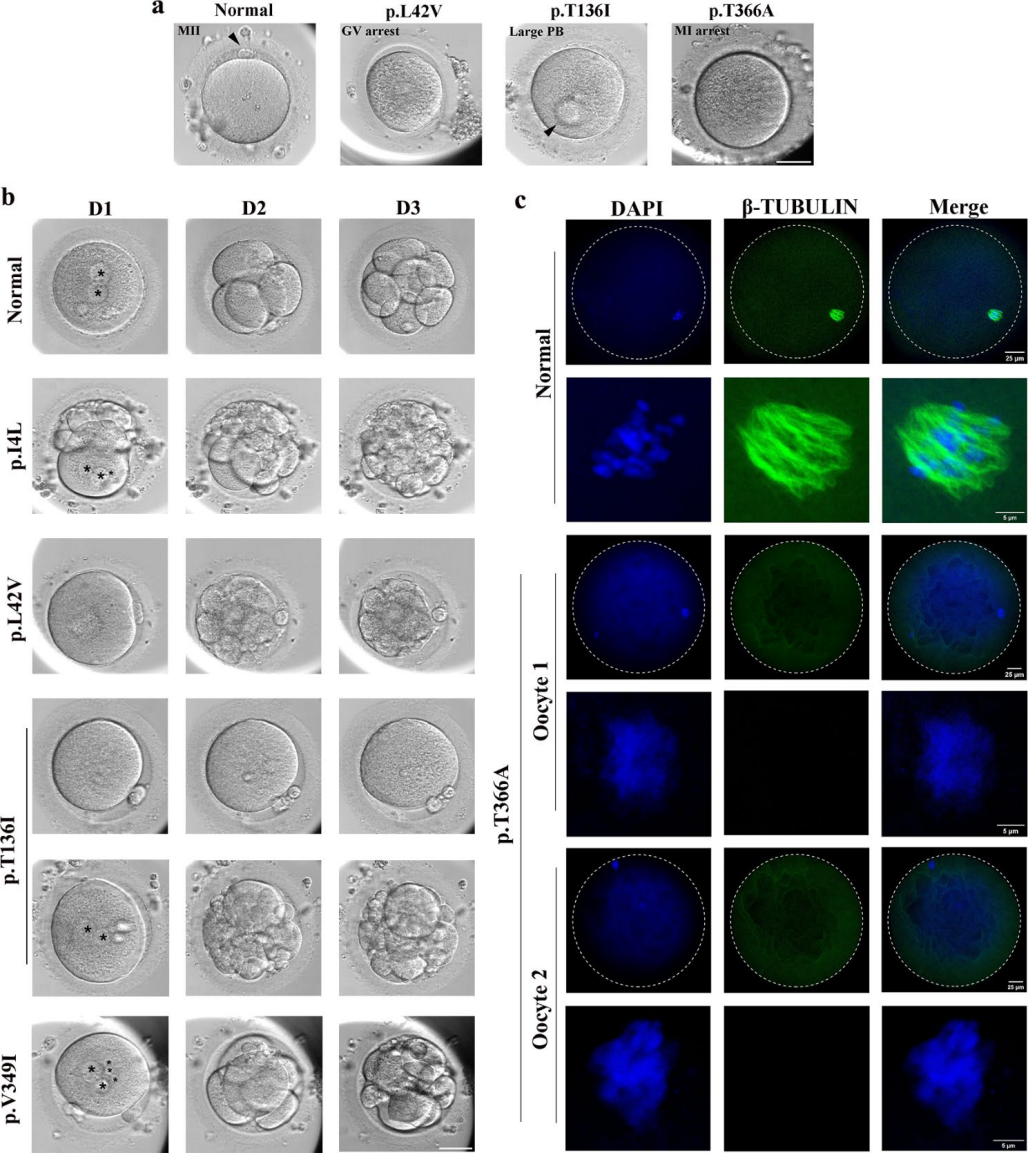
**Phenotypes of oocytes and embryos from probands with*TUBB8*variants. a** Morphology of oocytes from a normal control and three probands on day 0. The black arrows indicate polar bodies. Scale bar, 50 μm. **b** Morphology of zygotes on day 1, and embryos on day 2 and day 3 from a normal control and four probands. The black stars indicate pronucleus. Scale bar, 50 μm. **c** β-TUBULIN/DNA immunostaining of a normal MI oocyte and two MI oocytes form Family 9. Oocytes were immunoassayed with an antibody against β-TUBULIN (green) to visualize spindle and counterstained with DAPI (blue) to visualize DNA.

A detailed description of the clinical characteristics and outcomes of ART cycles of the nine affected females is presented in Table 2. The nine probands were aged between 26 and 35 years, suffered from primary infertility for 3 to 8 years, and had experienced 1–3 cycles of IVF or ICSI failure during ART treatment. Consistent with our previous findings [13], females harboring different *TUBB8* variants exhibited variable phenotypes in oocyte maturation and morphology, fertilization, cleavage, as well as early embryonic development. All oocytes obtained from the probands in Family 9 with p.T366A and in Family 4 with p.Y50C were arrested at GV or MI stage with no MII oocytes (Fig. 4a). For the probands with p.I4L, p.L42V or p.V349I variants in Family1, 3 and 8, 20-41.7% of the oocytes developed into MII stage but failed to be fertilized normally, presenting with no pronucleus or more than two pronuclei (Table 2; Fig. 4a). Some oocytes from the proband in Family 8 were with abnormal morphologies of large or more than one polar body. The most oocytes of 25 obtained from proband with p.T136I in Family 5 were at GV stage (n = 3), MI stage (n = 5), MII stage (n = 13), and with abnormal morphologies of large polar body (n = 4), respectively (Table 2; Fig. 4a). Only 38.5% (5/13) of the MII oocytes were successfully fertilized and cleaved normally, but developed into unusable embryos on day 3 (Fig. 4b). For proband in Family 6 with p.V255M variant, half of the retrieved 6 oocytes at MII stage were normally fertilized but with abnormal cleavage pattern (Table 2). Furthermore, probands in Family 2 with p.G13R variant and in Family 7 with compound heterozygous variants (p.F265V, p.Q134*) both had a high proportion of MII oocytes, 8 of 14 MII oocytes from proband with p.G13R were successfully fertilized, and 2 usable embryos were obtained. For proband with p.F265V and p.Q134*, 3 of 13 MII oocytes were successfully fertilized and 1 usable embryo was obtained. Unfortunately, both two probands failed to conceive after fresh embryo transfer (Table 2). Immunostaining of the MI oocyte from normal control showed that both spindle and DNA were visible, and the DNA was regularly arranged on both sides of the equatorial plate. In the MI oocytes from proband with p.T366A in Family 9, the spindles were invisible, and the DNA was disorderedly organized (Fig. 4c). Thus, we speculated that the *TUBB8* variants obstructed the oocyte maturation and early embryonic development by disrupting the normal function of spindle.

**Table 2:** Clinical characteristics of patients and their ART cycles

Family no.	Age (years)	Duration of infertility (years)	Previous ART cycles	Total no. of oocytes retrieved	GV oocyte	MI oocyte	MII oocyte	Oocyte with abnormal morphology	Degen- erated oocyte	Fertilized oocyte	Cleaved embryo	Usable embryo	Outcome of embryo transfer
Family 1	29	5	2	12	0	7	5	0	0	0	0	0	/
Family 2	34	7	3	15	1	0	14	0	0	8	7	2	Failure
Family 3	30	7	1	9	5	1	3	0	0	0	0	0	/
Family 4	35	8	1^a^	10	NA	NA	NA	NA	NA	NA	NA	0	/
			1	2	2	0	0	0	0	0	0	0	/
Family 5	26	7	2	25	3	5	13	4	0	5	5	0	/
Family 6	31	6	1	6	0	0	6	0	0	3	2	0	/
Family 7	30	7	1	13	0	0	13	0	0	3	1	1	Failure
Family 8	35	5	2	10	0	1	2	6	1	0	0	0	/
Family 9	29	3	1	6	1	5	0	0	0	0	0	0	/

ART, assisted reproductive technology; GV, germinal vesical; MI, metaphase I; MII, metaphase II; NA, not available

^a^ This ART cycle was conducted in other hospital

## Discussion

In this study, we recruited 35 females with primary infertility and identified ten different *TUBB8* variants in nine unrelated females, including nine missense variants (p.I4L, p.G13R, p.L42V, p.Y50C, p.T136I, p.V255M, p.F265V, p.V349I and p.T366A) and one loss-of-function variant (p.Q134*), respectively. These variants were inherited in dominant or recessive patterns, and even occurred *de novo*. Furthermore, we identified five novel variants and provided additional evidence for the clinical significance of five previously reported variants. The phenotypes caused by these variants were diverse, including abnormalities in oocyte maturation and morphology, fertilization failure, cleavage failure, embryonic development arrest and implantation failure. Through functional assay in vitro, we found that mutant TUBB8 proteins induced different degrees of microtubule structural disruption after exogenous expression in HeLa cells. These findings expanded the mutant spectrum of *TUBB8* gene and could facilitate the optimization of genetic counseling in primary infertile females.

So far, a total of 140 different variants in *TUBB8* gene have been reported, as summarized in Table S1. The vast majority of variant types in *TUBB8* were missense (87.9%, 123/140), and the rest were loss-of-function variants, including 14 frameshift (10%), 2 nonsense (1.4%) and 1 deletion (0.7%). The missense variants of *TUBB8* usually cause female infertility in a heterozygous pattern via dominant-negative effects, while loss-of-function variants are usually pathogenic when present in a compound heterozygous or homozygous pattern [15, 19, 23–28]. Consistent with previous studies, the novel *TUBB8* variants p.G13R, p.Y50C, p.T136I and p.T366A identified in this study presented in heterozygous pattern, implying their dominant-negative effects. For the compound heterozygous variant of p.F265V and p.Q134* in Family 7, given the pathogenic characteristics of the *TUBB8* loss-of-function variants mentioned above, we speculated that this compound heterozygous variant was *in trans* and confirmed it through TA cloning. It is worth noting that the phenotypes resulting from the heterozygous variant of p.F265V in *TUBB8* remains unknown.

Despite high heterogeneity is the hallmark of *TUBB8* variants, there are still some variants that have been reported repeatedly. Combining our present study with previously reported studies about *TUBB8* variants, we identified 5 candidate mutant-hotspots, namely p.I4L (n = 7), p.A313V (n = 7), p.V255M (n = 5), p.G98R (n = 5) and p.E108K (n = 5). Based on the reported studies we further reviewed the phenotypes of these candidate mutant-hotspots and found that the variants p.I4L and p.E108K appeared to affect the entire process from oocyte maturation to early embryonic development, accompanied by the phenotypes of GV arrest, MI arrest, oocytes with abnormal morphology, fertilization failure, cleavage failure and early embryonic development arrest [11, 13, 15, 19, 21, 22, 29]. The patients harboring variant p.A313V were reported to have conflicting ART results [16, 22, 30], nevertheless, our previous study showed that three patients with heterozygous p.A313V have good prognosis of ART outcomes and recent follow-up informed us that all of them had successful deliveries, so we speculated that it may be a benign variant [13]. The variant of p.V255M was reported to induce MI arrest in oocytes and early embryonic development arrest [17–19, 31], our study complemented the phenotypes of fertilization failure with p.V255M variant. The phenotypes have been reported for the variant p.G98R of *TUBB8* included MI arrest, fertilization failure and oocytes with abnormal morphology [11, 13, 19, 22, 32].

In this study, none of the females carrying *TUBB8* variants had successful pregnancy, neither by IVF nor ICSI. Through reviewing previously published studies, successful deliveries have only been reported in females with p.A313V and p.T429M [12, 13], implying the likely benign significance of these two variants, but it needs to be verified by more data. Thus far, there is no effective treatment strategies for female infertility caused by *TUBB8* variants, donor egg appears to be the most feasible treatment strategy for the females carrying pathogenic variants of *TUBB8*. Nevertheless, Jia et al. found that additional expression of wild-type TUBB8 cRNA in the mouse oocytes expressing mutant TUBB8 could rescue the embryonic development defects of resulting embryo and produce full-term offspring [33]. This study provides a possible means of treating female infertility caused by *TUBB8* variants and it seems to be a very promising direction for subsequent research.

## Conclusions

In conclusion, our study identified five novel variants to expand the mutant spectrum of *TUBB8* gene and described the phenotypes of five previously reported variants to strengthen the genotype–phenotype correlation of *TUBB8* variants. In light of the unfavorable reproductive outcomes in females harboring pathogenic *TUBB8* variants, genetic screen of *TUBB8* is of great importance to females diagnosed with primary infertility, and genetic counselling should be conducted cautiously.

## Methods

### Human subjects

From June 2022 to May 2023, 35 females diagnosed with primary infertility and experiencing IVF or ICSI failure due to disorders in oocyte maturation, fertilization, zygotic cleavage and early embryonic development from the Center for Reproductive Medicine, Women and Children’s Hospital of Chongqing Medical University (Chongqing, China) as well as the Reproductive Center, Northwest Women’s and Children’s Hospital (Xi’an, China.) were recruited. Through Sanger sequencing, a total of nine females harboring ten different *TUBB8* variants were finally enrolled in this study. A written informed consent was signed before the collection of blood or oocyte. Besides, the blood samples were collected from all females and their family members when possible.

### Sanger sequencing, TA cloning and variant analysis

Sanger sequencing was performed as previously reported [13]. Briefly, the amplicons of specific primers listed in Table S2 were sequenced with ABI 3500 (Thermo Fisher Scientific, Waltham, MA, USA) and analyzed with Chromas 2.6.5 (Technelysium Pvt. Ltd., South Brisbane, Australia). For TA cloning, the coding region of *TUBB8* were amplified via polymerase chain reaction, the amplicons were then cloned into pMD19T vector through TA cloning kit (TAKARA, Japan) according to the instruction. The gnomAD database (http://gnomad-sg.org/) was used to analyze the variants’ frequencies. The potential pathogenicity of all identified variants was evaluated by *in silico* analysis with Polymorphism Phenotyping v2 (PolyPhen-2, http://genetics.bwh.harvard.edu/pph2/index.shtml), Sorting Intolerant from Tolerant (SIFT, https://sift.bii.a-star.edu.sg/), MutationTaster (https://www.mutationtaster.org/), and Combined Annotation Dependent Depletion (CADD, https://cadd.gs.washington.edu/).

### Molecular modelling and amino acid conservation analysis

The diagram images of *TUBB8* gene and protein were modeled by Illustrator for Biological Sequences (IBS, http://ibs.biocuckoo.org/online.php). The amino acid conservation analysis of TUBB8 protein was performed among different species via the Clustal Omega tool (http://www.clustal.org/omega/). Wild-type and mutant TUBB8 protein structures were predicted using the SWISS-MODEL software (https://swissmodel.expasy.org) based on the 7rro.28.pdb template and further mapped to the atomic model using PyMol (http://www.pymol.org).

### Evaluation of oocytes and early embryos

The morphologies of retrieved oocytes were examined by light microscopy. Embryos were cultured after insemination using a time-lapse monitoring system (Embryoscope Plus, Vitrolife, Sweden) to limit exposure of embryos to suboptimal conditions. For the immunostaining, oocytes were first fixed in 4% paraformaldehyde for 30 min, then incubated in 0.5% Triton X-100 for 15 min to permeabilize the membrane and in 3% bovine serum albumin for 1 h to block. Oocytes were stained with an anti-β-TUBULIN FITC antibody (1:500 dilution, F2043, Sigma-Aldrich) and DAPI (P0131, Beyotime) to label the meiotic spindle and DNA, respectively. The images were captured on a confocal laser-scanning microscope (TCS SP8, Leica, Germany).

### Expression of WT and mutant TUBB8 proteins in HeLa cells

A full-length *TUBB8* cDNA cloned in a pCDNA3.1-Myc-HisA vector with a CMV promotor was purchased from Tsingke Biotechnology Co., Ltd. Point mutation using quick-change polymerase chain reaction was performed for the generation of TUBB8 mutants, including c.10 A > C, c.37G > A, c.124 C > G, c.149 A > G, c.400 C > T, c.407 C > T, c.763G > A, c.793T > G, c.1045G > A, c.1096 A > G. The plasmids expressing wild-type or mutant TUBB8 proteins were separately transfected in HeLa cells pre-seeded on 24-well plate by Lipofectamine 3000 (Thermo Fisher Scientific, USA) according to the manufacturer’s recommendations. After 48 h, the cells were fixed, permeabilized, and labeled with the antibodies against Myc epitope (1:500 dilution, AM926-1, Beyotime) to visualize transgene and α-TUBULIN (1:800 dilution, 5335s, CST) to visualize endogenous microtubule network. The next day, cells were incubated with Goat anti Rabbit IgG (H + L) Cross-Adsorbed Secondary Antibody, Alexa Fluor^TM^555 (1:500 dilution; A-21,428, Invitrogen, America) and Goat anti Mouse IgG (H + L) Cross-Adsorbed Secondary Antibody, Alexa Fluor^TM^488 (1:1000 dilution; A-11,001, Invitrogen, America). Cells were observed and photographed using confocal laser-scanning microscope (TCS SP8, Leica, Germany). For the quantification of microtubule phenotypes, approximately 200 cells expressing either wild-type or mutant TUBB8 were examined and classified according to the level of transgene expression (as judged by the fluorescence intensity) and microtubule structure (as judged by the microtubule appearance, filament density or diffuse mottled pattern of Myc and α-TUBULIN labels) in each of three independent experiments. In addition, in order to detect the expression level of wild-type and mutant proteins, the plasmids expressing wild-type or mutant TUBB8 were transfected in HeLa cells pre-seeded on 6-well plate. 48 h after transfection, the cells were collected and dissolved in RIPA lysis buffer (Beyotime, China) mixed with proteinase inhibitor to extract proteins, then the protein expression was detected by Western blot.

### Electronic supplementary material

Below is the link to the electronic supplementary material.

Supplementary Material 1

## Data Availability

The analyzed data generated during this study are available from the corresponding author on reasonable requests.

## List of abbreviations

ART: assisted reproductive technology
IVF: in vitro fertilization
ICSI: intracytoplasmic sperm injection
GV: germinal vesicle
MI: metaphase I
MII: metaphase II
OZEMA: oocyte/zygote/embryo maturation arrest
