# Supplementary material for: Novel variants in TUBB8 gene cause multiple phenotypic abnormalities in human oocytes and early embryos
Source: J Ovarian Res. 2023 Nov 25;16:228. doi: 10.1186/s13048-023-01274-3 (PMC10675859; doi:10.1186/s13048-023-01274-3)
Supplement: Supplementary file 1 — Supplementary Material 1 [file 13048_2023_1274_MOESM1_ESM.docx]

**Table S1. Summary of previously reported *TUBB8* variants in infertile females.**

| **Num** | **Gene** | **Transcript** | **cDNA Variation** | **Amino acid variation** | **Exon** | **Effect** | **Num of patients** | **Inheritance** | **Phenotype** | **Allelic status** | **Reference** |
| --- | --- | --- | --- | --- | --- | --- | --- | --- | --- | --- | --- |
| 1 | *TUBB8* | ENST00000357060 | c.5G>A | p.R2K | 1 | Missense | 1 | Father | MI arrest | Heterozygous | [1] |
| 2 | *TUBB8* | ENST00000357060 | c.527C>T | p.S176L | 4 | Missense | 1 | *De novo* | MI arrest | Heterozygous | [1] |
| 3 | *TUBB8* | ENST00000357060 | c.686T>C | p.V229A | 4 | Missense | 3 | Father | MI arrest | Heterozygous | [1] |
| 4 | *TUBB8* | ENST00000357060 | c.785G>A | p.R262Q | 4 | Missense | 1 | *De novo* | MI arrest | Heterozygous | [1] |
| 5 | *TUBB8* | ENST00000357060 | c.900G>A | p.M300I | 4 | Missense | 1 | Father | MI arrest | Heterozygous | [1] |
| 6 | *TUBB8* | ENST00000357060 | c.1088T>C | p.M363T | 4 | Missense | 1 | Father | MI arrest | Heterozygous | [1] |
| 7 | *TUBB8* | ENST00000357060 | c.1249G>A | p.D417N | 4 | Missense | 2 | Father | MI arrest | Heterozygous | [1] |
| 8 | *TUBB8* | ENST00000357060 | c.80_100del | p.E27_A33del | 2 | In-Frame del | 2 | Father/mother | MI arrest | Homozygous | [2] |
| 9 | *TUBB8* | ENST00000357060 | c.426dupG | p.T143Dfs*12 | 4 | Frameshift | 1 | Father/mother | MI arrest | Homozygous | [2] |
| 10 | *TUBB8* | ENST00000357060 | c.628A>G | p.I210V | 4 | Missense | 1 | Unknown | Early embryonic arrest | Heterozygous | [2] |
| 11 | *TUBB8* | ENST00000357060 | c.713C>T | p.T238M | 4 | Missense | 1 | Father | MI arrest | Heterozygous | [2] |
| 12 | *TUBB8* | ENST00000357060 | c.763G>A | p.V255M | 4 | Missense | 1 | *De novo* | MI arrest | Heterozygous | [2] |
| 13 | *TUBB8* | ENST00000357060 | c.784C>T | p.R262W | 4 | Missense | 1 | *De novo* | MI arrest | Heterozygous | [2] |
| 14 | *TUBB8* | ENST00000357060 | c.853A>C | p.T285P | 4 | Missense | 1 | Unknown | MI arrest | Heterozygous | [2] |
| 15 | *TUBB8* | ENST00000357060 | c.1043A>G | p.N348S | 4 | Missense | 1 | Unknown | MI arrest | Heterozygous | [2] |
| 16 | *TUBB8* | ENST00000357060 | c.5G>T | p.R2M | 1 | Missense | 1 | Father | MI arrest | Heterozygous | [3] |
| 17 | *TUBB8* | ENST00000357060 | c.10A>C | p.I4L | 1 | Missense | 1 | Father | MI arrest/Early embryonic arrest | Heterozygous | [3] |
| 18 | *TUBB8* | ENST00000357060 | c.35G>A | p.C12Y | 1 | Missense | 1 | Father/mother | MI arrest | Homozygous | [3] |
| 19 | *TUBB8* | ENST00000357060 | c.209C>T | p.P70L | 3 | Missense | 1 | Father/mother | Fertilization failure | Homozygous | [3] |
| 20 | *TUBB8* | ENST00000357060 | c.613G>A | p.E205K | 4 | Missense | 1 | Unknown | Immature oocyte (Unknown stage) | Heterozygous | [3] |
| 21 | *TUBB8* | ENST00000357060 | c.900G>A | p.M300I | 4 | Missense | 2 | Father | Early embryonic arrest | Heterozygous | [3] |
| 22 | *TUBB8* | ENST00000357060 | c.1057G>A | p.V353I | 4 | Missense | 1 | Unknown | MI arrest/Early embryonic arrest | Heterozygous | [3] |
| 23 | *TUBB8* | ENST00000357060 | c.580G>A | p.E194K | 4 | Missense | 1 | *De novo* | Immature oocyte (Unknown stage) | Compound heterozygous | [3] |
| 24 | *TUBB8* | ENST00000357060 | c.1245G>A | p.M415I | 4 | Missense |  |  |  |  |  |
| 25 | *TUBB8* | ENST00000357060 | Exon 1-4 deletion |  | 1-4 | Deletion | 1 | Father/mother | MI arrest | Homozygous | [3] |
| 26 | *TUBB8* | ENST00000357060 | c.5G>T | p.R2M | 1 | Missense | 1 | Father | MI arrest | Heterozygous | [4] |
| 27 | *TUBB8* | ENST00000357060 | c.535G>A | p.V179M | 4 | Missense | 2 | Father | MI arrest | Heterozygous | [4] |
| 28 | *TUBB8* | ENST00000357060 | c.292G>A | p.G98R | 4 | Missense | 1 | *De novo* | MI arrest | Heterozygous | [5] |
| 29 | *TUBB8* | ENST00000357060 | c.10A>C | p.I4L | 1 | Missense | 2 | *De novo*/Unknown | Fertilization failure/Early embryonic arrest | Heterozygous | [6] |
| 30 | *TUBB8* | ENST00000357060 | c.181C>A | p.P61T | 3 | Missense | 1 | *De novo* | Early embryonic arrest | Heterozygous | [6] |
| 31 | *TUBB8* | ENST00000357060 | c.292G>A | p.G98R | 4 | Missense | 2 | *De novo*/Unknown | MI arrest | Heterozygous | [6] |
| 32 | *TUBB8* | ENST00000357060 | c.322G>A | p.E108K | 4 | Missense | 1 | Father | Fertilization failure | Compound heterozygous | [6] |
| 33 | *TUBB8* | ENST00000357060 | c.426dupG | p.T143Dfs*12 | 4 | Frameshift |  | Mother |  |  | [6] |
| 34 | *TUBB8* | ENST00000357060 | c.523G>A | p.V175M | 4 | Missense | 1 | Unknown | MI arrest | Heterozygous | [6] |
| 35 | *TUBB8* | ENST00000357060 | c.527C>T | p.S176L | 4 | Missense | 2 | *De novo*/Unknown | MI arrest | Heterozygous | [6] |
| 36 | *TUBB8* | ENST00000357060 | c.600T>G | p.F200L | 4 | Missense | 1 | Unknown | Early embryonic arrest | Heterozygous | [6] |
| 37 | *TUBB8* | ENST00000357060 | c.721C>T | p.R241C | 4 | Missense | 1 | Father/mother | MI arrest | Homozygous | [6] |
| 38 | *TUBB8* | ENST00000357060 | c.722G>A | p.R241H | 4 | Missense | 1 | Unknown | MI arrest | Heterozygous | [6] |
| 39 | *TUBB8* | ENST00000357060 | c.735G>C | p.Q245H | 4 | Missense | 1 | *De novo* | Immature oocyte (Unknown stage) | Heterozygous | [6] |
| 40 | *TUBB8* | ENST00000357060 | c.763G>A | p.V255M | 4 | Missense | 6 | Father/Unknown | MI arrest/Early embryonic arrest | Heterozygous | [6] |
| 41 | *TUBB8* | ENST00000357060 | c.883G>C | p.D295H | 4 | Missense | 1 | Incomplete dominance | Early embryonic arrest | Heterozygous | [6] |
| 42 | *TUBB8* | ENST00000357060 | c.1000C>G | p.Q334E | 4 | Missense | 1 | Father | Early embryonic arrest | Heterozygous | [6] |
| 43 | *TUBB8* | ENST00000357060 | c.1057G>A | p.V353I | 4 | Missense | 1 | Unknown | Early embryonic arrest | Heterozygous | [6] |
| 44 | *TUBB8* | ENST00000357060 | c.1061G>A | p.C354Y | 4 | Missense | 1 | *De novo* | Immature oocyte (Unknown stage) | Heterozygous | [6] |
| 45 | *TUBB8* | ENST00000357060 | c.1073C>T | p.P358L | 4 | Missense | 5 | Father/*De novo*/Unknown | MI arrest | Heterozygous | [6] |
| 46 | *TUBB8* | ENST00000357060 | c.1072C>G | p.P358A | 4 | Missense | 1 | *De novo* | MI arrest | Heterozygous | [6] |
| 47 | *TUBB8* | ENST00000357060 | c.1099T>C | p.F367L | 4 | Missense | 1 | Father | MI arrest | Heterozygous | [6] |
| 48 | *TUBB8* | ENST00000357060 | c.1171C>T | p.R391C | 4 | Missense | 1 | Unknown | Early embryonic arrest | Heterozygous | [6] |
| 49 | *TUBB8* | ENST00000357060 | c.1205dupG | p.M403Hfs*3 | 4 | Frameshift | 1 | Father/mother | MI arrest | Homozygous | [6] |
| 50 | *TUBB8* | ENST00000357060 | c.1228G>A | p.E410K | 4 | Missense | 1 | *De novo* | Fertilization failure | Heterozygous | [6] |
| 51 | *TUBB8* | ENST00000357060 | c.1249G>T | p.D417Y | 4 | Missense | 1 | Unknown | Early embryonic arrest | Heterozygous | [6] |
| 52 | *TUBB8* | ENST00000357060 | c.1270C>T | p.Q424* | 4 | Nonsense | 1 | Father/mother | Fertilization failure | Homozygous | [6] |
| 53 | *TUBB8* | ENST00000357060 | c.1286C>T | p.T429M | 4 | Missense | 1 | Incomplete dominance | Unknown | Compound heterozygous | [6] |
| 54 | *TUBB8* | ENST00000357060 | c.1301_1327del | p.434_442del | 4 | In-frame del |  | Incomplete dominance |  |  |  |
| 55 | *TUBB8* | ENST00000357060 | c.322G>A | p.E108K | 4 | Missense | 1 | NA | Cleavage failure | Homozygous | [7] |
| 56 | *TUBB8* | ENST00000357060 | c.1054G > T | p.A352S | 4 | Missense | 2 | Unknown | MI arrest | Heterozygous | [8] |
| 57 | *TUBB8* | ENST00000357060 | c.1041C>A | p.N347K | 4 | Missense | 5 | Father | Early embryonic arrest | Heterozygous | [9] |
| 58 | *TUBB8* | ENST00000357060 | c.12C>G | p.I4M | 1 | Missense | 1 | Unknown | MI arrest | Heterozygous | [10] |
| 59 | *TUBB8* | ENST00000357060 | c.49G>A | p.G17S | 1 | Missense | 1 | Unknown | Immature oocyte (Unknown stage) | Heterozygous | [10] |
| 60 | *TUBB8* | ENST00000357060 | c.1103T>C | p.I368T | 4 | Missense | 1 | Unknown | MI arrest | Compound heterozygous | [10] |
| 61 | *TUBB8* | ENST00000357060 | c.80_100del | p.E27_A33del | 2 | In-frame del |  | Mother |  |  | [10] |
| 62 | *TUBB8* | ENST00000357060 | c.82C>T | p.H28Y | 2 | Missense | 1 | Father | Immature oocyte (Unknown stage) | Compound heterozygous | [10] |
| 63 | *TUBB8* | ENST00000357060 | c.148_154delinsCACCACCACGAGGCCAGCGGTGCGACCCCCGTCCTTCCCCCACCCAACGTGCACCACC | p.Y50_N52delinsHHHEASGATPVLPPPNVHHH | 2 | In-del |  | Mother |  |  | [10] |
| 64 | *TUBB8* | ENST00000357060 | c.398T>C | p.F133S | 4 | Missense | 1 | Unknown | Early embryonic arrest | Compound heterozygous | [10] |
| 65 | *TUBB8* | ENST00000357060 | c.82C>T | p.H28Y | 2 | Missense |  | Unknown |  |  | [10] |
| 66 | *TUBB8* | ENST00000357060 | c.148T>C | p.Y50H | 2 | Missense | 1 | Father | Oocytes with abnormal morphology | Heterozygous | [10] |
| 67 | *TUBB8* | ENST00000357060 | c.178G>C | p.V60L | 3 | Missense | 6 | Father | MI arrest | Heterozygous | [10] |
| 68 | *TUBB8* | ENST00000357060 | c.236G>A | p.G79E | 3 | Missense | 1 | Father | Early embryonic arrest | Heterozygous | [10] |
| 69 | *TUBB8* | ENST00000357060 | c.260C>T | P.P87L | 3 | Missense | 2 | Father | Immature oocyte (Unknown stage) | Heterozygous | [10] |
| 70 | *TUBB8* | ENST00000357060 | c.293G>A | p.G98E | 4 | Missense | 1 | Father | MI arrest | Heterozygous | [10] |
| 71 | *TUBB8* | ENST00000357060 | c.400C>T | p.Q134* | 4 | Nonsense | 1 | Mother | Immature oocyte (Unknown stage) | Compound heterozygous | [10] |
| 72 | *TUBB8* | ENST00000357060 | c.353A>G | p.D118G | 4 | Missense |  | Father |  |  | [10] |
| 73 | *TUBB8* | ENST00000357060 | c.1103T>C | p.I368T | 4 | Missense | 1 | Mother | MI arrest | Compound heterozygous | [10] |
| 74 | *TUBB8* | ENST00000357060 | c.382 dup | p.D128Gfs*27 | 4 | Frameshift |  | Father |  |  | [10] |
| 75 | *TUBB8* | ENST00000357060 | c.497C>T | p.T166I | 4 | Missense | 1 | Unknown | Immature oocyte (Unknown stage) | Heterozygous | [10] |
| 76 | *TUBB8* | ENST00000357060 | c.535G>C | p.V179L | 4 | Missense | 1 | Unknown | MI arrest | Heterozygous | [10] |
| 77 | *TUBB8* | ENST00000357060 | c.608A>G | p.D203G | 4 | Missense | 2 | Father | Immature oocyte (Unknown stage) | Heterozygous | [10] |
| 78 | *TUBB8* | ENST00000357060 | c.662C>T | p.T221I | 4 | Missense | 1 | Father | Oocytes with abnormal morphology | Heterozygous | [10] |
| 79 | *TUBB8* | ENST00000357060 | c.716G>C | p.C239S | 4 | Missense | 1 | *De novo* | Fertilization failure | Heterozygous | [10] |
| 80 | *TUBB8* | ENST00000357060 | c.728C>T | p.P243L | 4 | Missense | 2 | Unknown | Immature oocyte (Unknown stage) | Heterozygous | [10] |
| 81 | *TUBB8* | ENST00000357060 | c.728C>G | p.P243R | 4 | Missense | 1 | Unknown | Early embryonic arrest | Heterozygous | [10] |
| 82 | *TUBB8* | ENST00000357060 | c.736C>G | p.L246V | 4 | Missense | 2 | Unknown | MI arrest/Early embryonic arrest | Heterozygous | [10] |
| 83 | *TUBB8* | ENST00000357060 | c.743C>T | p.A248V | 4 | Missense | 1 | Father | Immature oocyte (Unknown stage) | Heterozygous | [10] |
| 84 | *TUBB8* | ENST00000357060 | c.845G>C | p.R282P | 4 | Missense | 1 | Unknown | MI arrest | Heterozygous | [10] |
| 85 | *TUBB8* | ENST00000357060 | c.893A>G | p.N298S | 4 | Missense | 1 | Father | Unknown | Heterozygous | [10] |
| 86 | *TUBB8* | ENST00000357060 | c.925C>T | p.R309C | 4 | Missense | 1 | Unknown | Immature oocyte (Unknown stage) | Heterozygous | [10] |
| 87 | *TUBB8* | ENST00000357060 | c.940G>T | p.A314S | 4 | Missense | 1 | Father | Early embryonic arrest | Heterozygous | [10] |
| 88 | *TUBB8* | ENST00000357060 | c.1076G>A | p.R359Q | 4 | Missense | 1 | Unknown | Immature oocyte (Unknown stage) | Homozygous | [10] |
| 89 | *TUBB8* | ENST00000357060 | c.1130T>C | p.L377P | 4 | Missense | 2 | Father | MI arrest/cleavage failure | Heterozygous | [10] |
| 90 | *TUBB8* | ENST00000357060 | c.1139G>A | p.R380H | 4 | Missense | 4 | Father/Unknown | Cleavage failure/Early embryonic arrest | Heterozygous | [10] |
| 91 | *TUBB8* | ENST00000357060 | c.1163T>C | p.M388T | 4 | Missense | 1 | *De novo* | MI arrest | Heterozygous | [10] |
| 92 | *TUBB8* | ENST00000357060 | c.1164G>A | p.M388I | 4 | Missense | 1 | Unknown | GV arrest | Heterozygous | [10] |
| 93 | *TUBB8* | ENST00000357060 | c.1172G>A | p.R391H | 4 | Missense | 7 | Father/Unknown | Immature oocyte (Unknown stage)/MI arrest/Early embryonic arrest | Heterozygous | [10] |
| 94 | *TUBB8* | ENST00000357060 | c.1187A>G | p.H396R | 4 | Missense | 1 | *De novo* | Early embryonic arrest | Heterozygous | [10] |
| 95 | *TUBB8* | ENST00000357060 | c.10_12delinsCTT | p.I4L | 1 | Missense | 1 | Father | MPN | Heterozygous | [11] |
| 96 | *TUBB8* | ENST00000357060 | c.524T>C | p.V175A | 4 | Missense | 1 | Father | MPN/Cleavage failure | Heterozygous | [11] |
| 97 | *TUBB8* | ENST00000357060 | c.1045G>A | p.V349I | 4 | Missense | 1 | Unknown | Early embryonic arrest | Heterozygous | [11] |
| 98 | *TUBB8* | ENST00000357060 | c.161C>T | p. A54V | 2 | Missense | 1 | Father/Mother | MI arrest | Homozygous | [12] |
| 99 | *TUBB8* | ENST00000357060 | c.735G>C | p.Q245H | 4 | Missense | 3 | Father | MI arrest | Heterozygous | [13] |
| 100 | *TUBB8* | ENST00000357060 | c.763G>A | p.V255M | 4 | Missense | 1 | *De novo* | MI arrest | Heterozygous | [13] |
| 101 | *TUBB8* | ENST00000357060 | c.845G>C | p.R282P | 4 | Missense | 1 | Unknown | MI arrest | Heterozygous | [13] |
| 102 | *TUBB8* | ENST00000357060 | c.608A>G | p.D203G | 4 | Missense | 1 | Father | Early embryonic arrest | Heterozygous | [14] |
| 103 | *TUBB8* | ENST00000357060 | c.817C>G | p.L273V | 4 | Missense | 1 | Unknown | Early embryonic arrest | Heterozygous | [14] |
| 104 | *TUBB8* | ENST00000357060 | c.161C>T | p. A54V | 2 | Missense | 2 | Father/Mother | MI arrest | Homozygous | [15] |
| 105 | *TUBB8* | ENST00000357060 | c.959G>A | p.R320H | 4 | Missense | 1 | Mother | Early embryonic arrest | Heterozygous | [15] |
| 106 | *TUBB8* | ENST00000357060 | c.208C>A | p.P70T | 3 | Missense | 1 | Unknown | Early embryonic arrest | Heterozygous | [16] |
| 107 | *TUBB8* | ENST00000357060 | c173G>A | p.R58K | 3 | Missense | 1 | Unknown | Early embryonic arrest | Heterozygous | [16] |
| 108 | *TUBB8* | ENST00000357060 | c.326G>T | p.G109V | 4 | Missense | 1 | Unknown | GV arrest | Heterozygous | [16] |
| 109 | *TUBB8* | ENST00000357060 | c.907T>C | p.C303R | 4 | Missense | 1 | Unknown | Early embryonic arrest | Heterozygous | [16] |
| 110 | *TUBB8* | ENST00000357060 | c.916C>T | p.R306C | 4 | Missense | 2 | Unknown | Early embryonic arrest | Compound heterozygous | [16] |
| 111 | *TUBB8* | ENST00000357060 | c.907T>C | p.C303R | 4 | Missense |  | Unknown |  |  | [16] |
| 112 | *TUBB8* | ENST00000357060 | c.10A>C | p.I4L | 1 | Missense | 1 | Unknown | Early embryonic arrest | Heterozygous | [17] |
| 113 | *TUBB8* | ENST00000357060 | c.136C>T | p.R46C | 2 | Missense | 1 | Unknown | Early embryonic arrest | Heterozygous | [17] |
| 114 | *TUBB8* | ENST00000357060 | c.292G>A | p.G98R | 4 | Missense | 2 | Unknown | MI arrest | Heterozygous | [17] |
| 115 | *TUBB8* | ENST00000357060 | c.367G>A | p.E123K | 4 | Missense | 1 | Unknown | MI arrest | Heterozygous | [17] |
| 116 | *TUBB8* | ENST00000357060 | c.394G>T | p.G132C | 4 | Missense | 1 | Unknown | MI arrest | Heterozygous | [17] |
| 117 | *TUBB8* | ENST00000357060 | c.422G>C | p.G141A | 4 | Missense | 1 | Unknown | MI arrest | Heterozygous | [17] |
| 118 | *TUBB8* | ENST00000357060 | c.527C>T | p.S176L | 4 | Missense | 1 | Unknown | MI arrest | Heterozygous | [17] |
| 119 | *TUBB8* | ENST00000357060 | c.535G>A | p.V179M | 4 | Missense | 3 | Unknown | MI arrest | Heterozygous | [17] |
| 120 | *TUBB8* | ENST00000357060 | c.539T>C | p.V180A | 4 | Missense | 1 | Unknown | MI arrest | Heterozygous | [17] |
| 121 | *TUBB8* | ENST00000357060 | c.544C>T | p.P182S | 4 | Missense | 1 | Unknown | MI arrest and fertilization failure | Heterozygous | [17] |
| 122 | *TUBB8* | ENST00000357060 | c.550A>G | p.N184D | 4 | Missense | 1 | Unknown | MI arrest | Homozygous | [17] |
| 123 | *TUBB8* | ENST00000357060 | c.594G>C | p.E198D | 4 | Missense | 1 | Father | MI arrest and fertilization failure | Heterozygous | [17] |
| 124 | *TUBB8* | ENST00000357060 | c.613G>A | p.E205K | 4 | Missense | 1 | Unknown | MPN | Heterozygous | [17] |
| 125 | *TUBB8* | ENST00000357060 | c.629T>A | p.I210K | 4 | Missense | 1 | Unknown | Fertilization failure | Heterozygous | [17] |
| 126 | *TUBB8* | ENST00000357060 | c.629T>A | p.I210K | 4 | Missense | 1 | *De novo* | MI arrest and fertilization failure | Heterozygous | [17] |
| 127 | *TUBB8* | ENST00000357060 | c.713C>T | p.T238M | 4 | Missense | 1 | Father | MI arrest | Heterozygous | [17] |
| 128 | *TUBB8* | ENST00000357060 | c.722G>A | p.R241H | 4 | Missense | 1 | Unknown | MI arrest | Heterozygous | [17] |
| 129 | *TUBB8* | ENST00000357060 | c.728C>T | p.P243L | 4 | Missense | 1 | Unknown | MI arrest | Heterozygous | [17] |
| 130 | *TUBB8* | ENST00000357060 | c.893A>G | p.N298S | 4 | Missense | 1 | Father | MI arrest and early embryonic arrest | Heterozygous | [17] |
| 131 | *TUBB8* | ENST00000357060 | c.938C>T | p.A313V | 4 | Missense | 1 | Unknown | Fertilization failure | Heterozygous | [17] |
| 132 | *TUBB8* | ENST00000357060 | c.10A>C | p.I4L | 1 | Missense | 1 | Unknown | Early embryonic arrest | Compound heterozygous | [17] |
| 133 | *TUBB8* | ENST00000357060 | c.938C>T | p.A313V | 4 | Missense |  | Unknown |  |  | [17] |
| 134 | *TUBB8* | ENST00000357060 | c.940G>T | p.A314S | 4 | Missense | 1 | Unknown | Fertilization failure and early embryonic arrest | Heterozygous | [17] |
| 135 | *TUBB8* | ENST00000357060 | c.1045G>A | p.V349I | 4 | Missense | 1 | Unknown | Early embryonic arrest | Heterozygous | [17] |
| 136 | *TUBB8* | ENST00000357060 | c.1073C>T | p.P358L | 4 | Missense | 1 | Father | MI arrest | Heterozygous | [17] |
| 137 | *TUBB8* | ENST00000357060 | c.1130T>C | p.L377P | 4 | Missense | 1 | Unknown | Fertilization failure | Heterozygous | [17] |
| 138 | *TUBB8* | ENST00000357060 | c.1139G>A | p.R380H | 4 | Missense | 1 | Unknown | MI arrest and fertilization failure | Heterozygous | [17] |
| 139 | *TUBB8* | ENST00000357060 | c.1163T>C | p.M388T | 4 | Missense | 1 | Unknown | MI arrest | Heterozygous | [17] |
| 140 | *TUBB8* | ENST00000357060 | c.1172G>A | p.R391H | 4 | Missense | 1 | Unknown | MI arrest | Heterozygous | [17] |
| 141 | *TUBB8* | ENST00000357060 | c.1178C>A | p.A393D | 4 | Missense | 1 | Father | MI arrest | Heterozygous | [17] |
| 142 | *TUBB8* | ENST00000357060 | c.1189T>G | p.W397G | 4 | Missense | 1 | Unknown | MI arrest | Heterozygous | [17] |
| 143 | *TUBB8* | ENST00000357060 | c.1203_1204insCT | p.G402Lfs*15 | 4 | Frameshift | 1 | Unknown | Fertilization failure | Homozygous | [17] |
| 144 | *TUBB8* | ENST00000357060 | c.1242C>G | p.N414K | 4 | Missense | 1 | *De novo* | MI arrest | Heterozygous | [17] |
| 145 | *TUBB8* | ENST00000357060 | c.1271A>G | p.Q424R | 4 | Missense | 1 | Unknown | MI arrest | Heterozygous | [17] |
| 146 | *TUBB8* | ENST00000357060 | c.6G>T | p.R2S | 1 | Missense | 2 | Father | Early embryonic arrest | Heterozygous | [18] |
| 147 | *TUBB8* | ENST00000357060 | c.43C>A | p.Q15K | 1 | Missense | 1 | Father | Early embryonic arrest | Heterozygous | [18] |
| 148 | *TUBB8* | ENST00000357060 | c.124C>G | p.L42V | 2 | Missense | 1 | Unknown | Early embryonic arrest | Heterozygous | [18] |
| 149 | *TUBB8* | ENST00000357060 | c.262G>C | p.D88H | 3 | Missense | 1 | Unknown | Early embryonic arrest | Heterozygous | [18] |
| 150 | *TUBB8* | ENST00000357060 | c.269dupT | p.I91Hfs*35 | 3 | Frameshift | 1 | Unknown | Cleavage failure | Compound heterozygous | [18] |
| 151 | *TUBB8* | ENST00000357060 | c.426dupG | p.T143Dfs*12 | 4 | Frameshift |  | Father |  |  | [18] |
| 152 | *TUBB8* | ENST00000357060 | c.292G>A | p.G98R | 4 | Missense | 1 | *De novo* | Fertilization failure | Heterozygous | [18] |
| 153 | *TUBB8* | ENST00000357060 | c.322G>A | p.E108K | 4 | Missense | 1 | Unknown | Cleavage failure | Homozygous | [18] |
| 154 | *TUBB8* | ENST00000357060 | c.322G>A | p.E108K | 4 | Missense | 1 | Father | Cleavage failure | Compound heterozygous | [18] |
| 155 | *TUBB8* | ENST00000357060 | c.966dupC | p.M323Hfs*6 | 4 | Frameshift |  | Mother |  |  | [18] |
| 156 | *TUBB8* | ENST00000357060 | c.421G>C | p.G141R | 4 | Missense | 1 | Unknown | Early embryonic arrest | Heterozygous | [18] |
| 157 | *TUBB8* | ENST00000357060 | c.544C>T | p.P182S | 4 | Missense | 1 | Father | Early embryonic arrest | Heterozygous | [18] |
| 158 | *TUBB8* | ENST00000357060 | c.586G>A | p.A196T | 4 | Missense | 1 | Mother | Early embryonic arrest | Heterozygous | [18] |
| 159 | *TUBB8* | ENST00000357060 | c.604A>C | p.I202L | 4 | Missense | 1 | Father | Early embryonic arrest | Heterozygous | [18] |
| 160 | *TUBB8* | ENST00000357060 | c.694A>T | p.T232S | 4 | Missense | 1 | Mother | Early embryonic arrest | Heterozygous | [18] |
| 161 | *TUBB8* | ENST00000357060 | c.722G>C | p.R241P | 4 | Missense | 1 | Mother | Oocyte meiotic arrest | Compound heterozygous | [18] |
| 162 | *TUBB8* | ENST00000357060 | c.1190_1192dup | p.W397dup | 4 | In-frame dup |  | Father |  |  | [18] |
| 163 | *TUBB8* | ENST00000357060 | c.785G>A | p.R262Q | 4 | Missense | 2 | *De novo* | MI arrest/cleavage failure | Heterozygous | [18] |
| 164 | *TUBB8* | ENST00000357060 | c.898A>T | p.M300L | 4 | Missense | 1 | Father | Early embryonic arrest | Heterozygous | [18] |
| 165 | *TUBB8* | ENST00000357060 | c.904G>A | p.A302T | 4 | Missense | 1 | Father | Early embryonic arrest | Heterozygous | [18] |
| 166 | *TUBB8* | ENST00000357060 | c.922G>A | p.G308S | 4 | Missense | 2 | Father/*De novo* | Cleavage failure | Homozygous | [18] |
| 167 | *TUBB8* | ENST00000357060 | c.959G>A | p.R320H | 4 | Missense | 2 | Mother | Early embryonic arrest | Heterozygous | [18] |
| 168 | *TUBB8* | ENST00000357060 | c.1055C>T | p.A352V | 4 | Missense | 1 | *De novo* | Early embryonic arrest | Heterozygous | [18] |
| 169 | *TUBB8* | ENST00000357060 | c.1057G>A | p.V353I | 4 | Missense | 2 | Father/*De novo* | Cleavage failure | Heterozygous | [18] |
| 170 | *TUBB8* | ENST00000357060 | c.1072C>T | p.P358S | 4 | Missense | 1 | Unknown | Cleavage failure | Heterozygous | [18] |
| 171 | *TUBB8* | ENST00000357060 | c.1139G>A | p.R380H | 4 | Missense | 1 | Unknown | Oocyte meiotic arrest | Heterozygous | [18] |
| 172 | *TUBB8* | ENST00000357060 | c.1171C>T | p.R391C | 4 | Missense | 2 | *De novo* | Early embryonic arrest | Heterozygous | [18] |
| 173 | *TUBB8* | ENST00000357060 | c.1172G>T | p.R391L | 4 | Missense | 1 | *De novo* | Early embryonic arrest | Heterozygous | [18] |
| 174 | *TUBB8* | ENST00000357060 | c.1172G>A | p.R391H | 4 | Missense | 1 | Unknown | Early embryonic arrest | Heterozygous | [18] |
| 175 | *TUBB8* | ENST00000357060 | c.1232C>A | p.A411D | 4 | Missense | 1 | Mother | Early embryonic arrest | Heterozygous | [18] |
| 176 | *TUBB8* | ENST00000357060 | c.286G>A | p.G96R | 4 | Missense | 1 | Unknown | MI arrest | Heterozygous | [19] |
| 177 | *TUBB8* | ENST00000357060 | c.717C>G | p.C239W | 4 | Missense | 1 | *De novo* | MI arrest | Heterozygous | [19] |
| 178 | *TUBB8* | ENST00000357060 | c.752G>A | p.R251Q | 4 | Missense | 1 | Unknown | MI arrest | Homozygous | [19] |
| 179 | *TUBB8* | ENST00000357060 | c. 938C>T | p.A313V | 4 | Missense | 1 | Unknown | MI arrest | Heterozygous | [19] |
| 180 | *TUBB8* | ENST00000357060 | c.1073C>T | p.P358L | 4 | Missense | 1 | Unknown | MI arrest | Heterozygous | [19] |
| 181 | *TUBB8* | ENST00000357060 | c.898_900del | p.M300del | 4 | In-frame del | 1 | Father | MI arrest | Heterozygous | [20] |
| 182 | *TUBB8* | ENST00000357060 | c.938C>T | p.A313V | 4 | Missense | 1 | Unknown | Recurrent pregnancy loss | Heterozygous | [20] |
| 183 | *TUBB8* | ENST00000357060 | c.535G>A | p.V179M | 4 | Missense | 2 | Unknown | MI arrest | Heterozygous | [21] |
| 184 | *TUBB8* | ENST00000357060 | c.686T>C | p.V229A | 4 | Missense | 1 | Unknown | MI arrest | Heterozygous | [21] |
| 185 | *TUBB8* | ENST00000357060 | c.527C>G | p.S176W | 4 | Missense | 1 | Unknown | MI arrest | Heterozygous | [21] |
| 186 | *TUBB8* | ENST00000357060 | c.763G>A | p.V255M | 4 | Missense | 1 | Unknown | MI arrest | Heterozygous | [21] |
| 187 | *TUBB8* | ENST00000357060 | c.538G>A | p.V180M | 4 | Missense | 2 | Father | MI arrest | Heterozygous | [22] |
| 188 | *TUBB8* | ENST00000357060 | c.527C>G | p.S176W | 4 | Missense | 1 | Unknown | MI arrest and Oocytes with abnormal morphology | Heterozygous | [22] |
| 189 | *TUBB8* | ENST00000357060 | c.124 C>G | p.L42V | 2 | Missense | 1 | Unknown | GV arrest and MI arrest | Heterozygous | [22] |
| 190 | *TUBB8* | ENST00000357060 | c.628A>C | p.I210L | 4 | Missense | 1 | Unknown | MI arrest/ Oocytes with abnormal morphology / fertilization failure | Heterozygous | [22] |
| 191 | *TUBB8* | ENST00000357060 | c.730G>A | p.G244S | 4 | Missense | 1 | Father | MI arrest | Heterozygous | [23] |
| 192 | *TUBB8* | ENST00000357060 | c.124 C>G | p.L42V | 2 | Missense | 1 | Unknown | Unknown | Heterozygous | [23] |
| 193 | *TUBB8* | ENST00000357060 | c.178G>A | p.V60M | 3 | Missense | 1 | *De novo* | MI arrest | Heterozygous | [23] |
| 194 | *TUBB8* | ENST00000357060 | c.1172G>T | p.R391L | 4 | Missense | 1 | *De novo* | GV arrest and MI arrest | Heterozygous | [23] |
| 195 | *TUBB8* | ENST00000357060 | c.10A>C | p.I4L | 1 | Missense | 1 | Unknown | GV arrest/MI arrest/ Oocytes with abnormal morphology | Heterozygous | [24] |
| 196 | *TUBB8* | ENST00000357060 | c.292G>A | p.G98R | 4 | Missense | 1 | Father | Oocytes with abnormal morphology | Heterozygous | [24] |
| 197 | *TUBB8* | ENST00000357060 | c.322G>A | p.E108K | 4 | Missense | 1 | Father | GV arrest/MI arrest/ Oocytes with abnormal morphology/ Early embryonic arrest | Heterozygous | [24] |
| 198 | *TUBB8* | ENST00000357060 | c.538dup | p.V180Gfs*19 | 4 | Frameshift | 1 | Father&mother | MI arrest | Homozygous | [24] |
| 199 | *TUBB8* | ENST00000357060 | c.563_564del | p.S188Cfs*10 | 4 | Frameshift | 1 | Unknown | Natural pregnant | Heterozygous | [24] |
| 200 | *TUBB8* | ENST00000357060 | c.673C>G | p.L225V | 4 | Missense | 1 | *De novo* | Oocytes with abnormal morphology/ Early embryonic arrest | Heterozygous | [24] |
| 201 | *TUBB8* | ENST00000357060 | c.784C>T | p.R262W | 4 | Missense | 1 | Unknown | Oocytes with abnormal morphology/ Early embryonic arrest | Heterozygous | [24] |
| 202 | *TUBB8* | ENST00000357060 | c.938C>T | p.A313V | 4 | Missense | 1 | Mother | Clinical pregnancy | Heterozygous | [24] |
| 203 | *TUBB8* | ENST00000357060 | c.938C>T | p.A313V | 4 | Missense | 1 | Unknown | Fertilization failure and Biochemical pregnancy | Heterozygous | [24] |
| 204 | *TUBB8* | ENST00000357060 | c.938C>T | p.A313V | 4 | Missense | 1 | Mother | Early embryonic arrest | Heterozygous | [24] |
| 205 | *TUBB8* | ENST00000357060 | c.1138C>T | p.R380C | 4 | Missense | 1 | Mother | Oocytes with abnormal morphology/ fertilization failure/ Early embryonic arrest | Heterozygous | [24] |
| 206 | *TUBB8* | ENST00000357060 | c.1138C>T | p.R380C | 4 | Missense | 1 | Father | Oocytes with abnormal morphology/ fertilization failure/ Early embryonic arrest | Heterozygous | [24] |
| 207 | *TUBB8* | ENST00000357060 | c.1139G>A | p.R380H | 4 | Missense | 1 | Father | MI arrest/ Oocytes with abnormal morphology /Cleavage failure | Heterozygous | [24] |
| 208 | *TUBB8* | ENST00000357060 | c.1172G>A | p.R391H | 4 | Missense | 1 | Father | MI arrest and fertilization failure | Heterozygous | [24] |
| 209 | *TUBB8* | ENST00000357060 | c.915_916del | p.R306Sfs*21 | 4 | Frameshift | 1 | Unknown | Early embryonic arrest | Compound heterozygous | [25] |
| 210 | *TUBB8* | ENST00000357060 | c.82C>T | p.H28Y | 2 | Missense |  | Unknown |  |  | [25] |
| 211 | *TUBB8* | ENST00000357060 | c.1286C>T | p.T429M | 4 | Missense | 1 | Father | Early embryonic arrest, delivered after 2nd ET. | Heterozygous | [25] |

**Reference**

1. Feng R, Sang Q, Kuang Y, Sun X, Yan Z, Zhang S, et al. Mutations in TUBB8 and Human Oocyte Meiotic Arrest. N Engl J Med. 2016;374:223–32.
2. Feng R, Yan Z, Li B, Yu M, Sang Q, Tian G, et al. Mutations in TUBB8 cause a multiplicity of phenotypes in human oocytes and early embryos. J Med Genet. 2016;53:662–71.
3. Chen B, Li B, Li D, Yan Z, Mao X, Xu Y, et al. Novel mutations and structural deletions in TUBB8 : expanding mutational and phenotypic spectrum of patients with arrest in oocyte maturation, fertilization or early embryonic development. Hum Reprod. 2017;32:457–64.
4. Huang L, Tong X, Luo L, Zheng S, Jin R, Fu Y, et al. Mutation analysis of the TUBB8 gene in nine infertile women with oocyte maturation arrest. Reproductive BioMedicine Online. 2017;35:305–10.
5. Wang A-C, Zhang Y-S, Wang B-S, Zhao X-Y, Wu F-X, Zhai X-H, et al. Mutation analysis of the TUBB8 gene in primary infertile women with arrest in oocyte maturation. Gynecological Endocrinology. 2018;34:900–4.
6. Chen B, Wang W, Peng X, Jiang H, Zhang S, Li D, et al. The comprehensive mutational and phenotypic spectrum of TUBB8 in female infertility. Eur J Hum Genet. 2019;27:300–7.
7. Yuan P, Zheng L, Liang H, Li Y, Zhao H, Li R, et al. A novel mutation in the TUBB8 gene is associated with complete cleavage failure in fertilized eggs. J Assist Reprod Genet. 2018;35:1349–56.
8. Xiang J, Wang W, Qian C, Xue J, Wang T, Li H, et al. Human oocyte maturation arrest caused by a novel missense mutation in TUBB8. J Int Med Res. 2018;46:3759–64.
9. Jia Y, Li K, Zheng C, Tang Y, Bai D, Yin J, et al. Identification and rescue of a novel TUBB8 mutation that causes the first mitotic division defects and infertility. J Assist Reprod Genet. 2020;37:2713–22.
10. Zhao L, Guan Y, Wang W, Chen B, Xu S, Wu L, et al. Identification novel mutations in TUBB8 in female infertility and a novel phenotype of large polar body in oocytes with TUBB8 mutations. J Assist Reprod Genet. 2020;37:1837–47.
11. Sha Q, Zheng W, Feng X, Yuan R, Hu H, Gong F, et al. Novel mutations in TUBB8 expand the mutational and phenotypic spectrum of patients with zygotes containing multiple pronuclei. Gene. 2021;769:145227.
12. Xing Q, Wang R, Chen B, Li L, Pan H, Li T, et al. Rare homozygous mutation in TUBB8 associated with oocyte maturation defect-2 in a consanguineous mating family. J Ovarian Res. 2020;13:42.
13. Lanuza-López MC, Martínez-Garza SG, Solórzano-Vázquez JF, Paz-Cervantes D, González-Ortega C, Maldonado-Rosas I, et al. Oocyte maturation arrest produced by TUBB8 mutations: impact of genetic disorders in infertility treatment. Gynecological Endocrinology. 2020;36:829–34.
14. Liu Z, Xi Q, Zhu L, Yang X, Jin L, Wang J, et al. TUBB8 Mutations Cause Female Infertility with Large Polar Body Oocyte and Fertilization Failure. Reprod Sci. 2021;28:2942–50.
15. Cao T, Guo J, Xu Y, Lin X, Deng W, Cheng L, et al. Two mutations in TUBB8 cause developmental arrest in human oocytes and early embryos. Reproductive BioMedicine Online. 2021;43:891–8.
16. Lu Q, Zhang X, Cao Q, Wang C, Ding J, Zhao C, et al. Expanding the Genetic and Phenotypic Spectrum of Female Infertility Caused by TUBB8 Mutations. Reprod Sci. 2021;28:3448–57.
17. Yang P, Yin C, Li M, Ma S, Cao Y, Zhang C, et al. Mutation analysis of tubulin beta 8 class VIII in infertile females with oocyte or embryonic defects. Clinical Genetics. 2021;99:208–14.
18. Zheng W, Hu H, Zhang S, Xu X, Gao Y, Gong F, et al. The comprehensive variant and phenotypic spectrum of TUBB8 in female infertility. J Assist Reprod Genet. 2021;38:2261–72.
19. Yao Z, Zeng J, Zhu H, Zhao J, Wang X, Xia Q, et al. Mutation analysis of the TUBB8 gene in primary infertile women with oocyte maturation arrest. J Ovarian Res. 2022;15:38.
20. Yuan H, Chen J, Li N, Miao H, Chen Y, Lyu S, et al. Target-Sequencing of Female Infertility Pathogenic Gene Panel and a Novel TUBB8 Loss-of-Function Mutation. Front Genet. 2022;13:865103.
21. Huo M, Zhang Y, Shi S, Shi H, Liu Y, Zhang L, et al. Gene Spectrum and Clinical Traits of Nine Patients With Oocyte Maturation Arrest. Front Genet. 2022;13:772143.
22. Yu W, Zhang S, Yin B, Dong C, Zhang VW, Zhang C. Identification of TUBB8 Variants in 5 Primary Infertile Women with Multiple Phenotypes in Oocytes and Early Embryos. Reprod Sci. 2023;30:1376–82.
23. Li W, Li Q, Xu X, Wang C, Hu K, Xu J. Novel mutations in TUBB8 and ZP3 cause human oocyte maturation arrest and female infertility. European Journal of Obstetrics & Gynecology and Reproductive Biology. 2022;279:132–9.
24. Lin T, Liu W, Han W, Tong K, Xiang Y, Liao H, et al. Genetic screening and analysis of TUBB8 variants in females seeking ART. Reproductive BioMedicine Online. 2023;46:244–54.
25. Zhang J, Li S, Huang F, Xu R, Wang D, Song T, et al. A novel compound heterozygous mutation in TUBB8 causing early embryonic developmental arrest. J Assist Reprod Genet [Internet]. 2023 [cited 2023 May 17]; Available from: https://link.springer.com/10.1007/s10815-023-02734-x.

**Table S2. The primers for PCR reactions and Sanger sequencing.**

| **Gene** | **Region** | **Primer** | **Sequence (5' to 3')** | **Product size** |
| --- | --- | --- | --- | --- |
| *TUBB8* | Exon 1-3 | Forward | CCTGAGCTTTCCAACAGATGTTCA | 918 bp |
|  |  | Reverse | CCCAGAGGATGACCTTAGCA |  |
|  | Exon 4 | Forward | AGGTGAGGAGTTACTGATGTAAAC | 2540 bp |
|  |  | Reverse | GGAGAACACTGTCCGTGCAT |  |

PCR, polymerase chain reaction.
